# Reliability and Agreement of the 10-Repetition Maximum Test in Breast Cancer Survivors

**DOI:** 10.3389/fonc.2019.00918

**Published:** 2019-09-26

**Authors:** Wanderson Divino Nilo dos Santos, Gabriel Dutra de Jesus Siqueira, Wagner Rodrigues Martins, Amilton Vieira, Raquel Machado Schincaglia, Paulo Gentil, Carlos Alexandre Vieira

**Affiliations:** ^1^College of Physical Education and Dance, Federal University of Goias - UFG, Goiânia, Brazil; ^2^College of Physical Education, University of Brasilia - UnB, Brasilia, Brazil; ^3^College of Nutrition, Federal University of Goias - UFG, Goiânia, Brazil

**Keywords:** muscle strength, muscular measurement, strength training, resistance training, cancer

## Abstract

The aim of this study was to evaluate the reliability and agreement between the test and retest of the 10-repetition maximum (10-RM) test for leg press and bench press in breast cancer survivors (BCS). Thirty-one BCS participated in this study, age 54.87 ± 5.7 years. All performed 10-RM tests and retests for the leg press 45° and the bench press. For reliability analyses, an intraclass coefficient correlation (ICC) and coefficient of variation (CV) were performed. The limits of agreement were calculated using a Bland-Altman plot with 95% CIs. For absolute and relative error of measurement, we used standard error of measurement and minimally detectable change. The result showed a high reliability for the bench press and leg press; ICC of 0.94 and 0.98, respectively. CV was <10% for both exercises. The systematic error were 1.5 kg (10%) and 6.1 (8%) for the bench press and leg press, respectively. The standard errors of measurements were 0.96 kg (6.08%) and 4.11 kg (5.27%) for the bench press and leg press, respectively. The minimally detectable changes were 2.72 kg (17.20%) and 5.62 kg (7.21%) for the bench press and leg press, respectively. In breast cancer survivors, the muscular strength measurement for the 10-RM test showed a high to very high rate of reliability and agreement, with acceptable error of measurement.

## Introduction

The assessment of muscle strength has been used to monitor and prescribe strength training (1). Muscular strength has been associated with high level of functional capacity and to decrease the risk of death from all natural causes (2, 3). The evaluation of muscle strength in breast cancer survivors (BCS) is a significant issue, because breast cancer (BC) treatment could reduce muscle strength after surgery and it may persist over the long-term (4). Therefore, in rehabilitation settings, the assessment of muscular strength is an important strategy to guide exercise prescription in these patients (5, 6).

Muscular strength loss in BCS is one of the side effects of BC treatment (surgery, chemotherapy, and radiotherapy), that could be explained by multiple factors such as: fatigue; lymphedema; decreased in shoulder, elbow, and wrist mobility; pain in the shoulder joint; and psychological changes such as kinesiophobia (7–13). These conditions could interfere with the reliability of maximum force tests and the strength outcomes during resistance training (14). In addition, these side effects of BC treatment pose a challenge for health professionals who work with resistance training for BC patients or survivors.

The one-repetition maximum test (1-RM) is considered the “gold standard” to measure maximum muscle strength in a non-laboratory setting. The 1-RM test is safe and has been applied in studies with BCS and BC patients (15–17). However, there is a lack of data regarding the reliability of this measurement within this population. To our knowledge, a single study presented only the coefficient of variation (CV) data for the bench press and leg press (18). Moreover, the 1-RM receive some criticism during a rehabilitation scenario such as risk of injury (19).

As an alternative to the 1-RM test, some studies with BCS used predictive formulas according to the results of multiple repetition tests (5-10RM) to estimate the maximum strength by 1-RM (20–23). Another method to estimate dynamic muscle strength is the repetition maximum test based on a goal of repetitions, as in the 10-RM test. The 10-RM test has been used to evaluate the load achieved in resistance training (RT) in different populations (24–30). Therefore, taking into consideration the characteristics of BCS, it seems that there is a natural concern with muscular strength tests for upper limbs, and maybe that could interfere on reliability of measurement. For this reason, it is possible that muscular strength for lower limbs could be more reliable than upper limbs. In addition, there is little information on the data of reliability and agreement of muscular strength tests in BCS, thus the performance of reliability studies is necessary.

The objective of this study was therefore to evaluate the reliability and agreement between the test and retest of the 10-RM test in upper and lower limbs in BCS. Our hypothesis was that the 10-RM test is reliable, and that the reliability is higher for the lower limbs.

## Materials and Methods

### Design and Participants

In this reliability and agreement study, 31 BCS were included between February and October 2017. The BCS were contacted via phone calls and face-to-face interactions at the Mastology and Oncology Ambulatory of the University Hospital of the Federal University of Goias, Brazil. The eligibility criteria were: (1) confirmed BC stages I to III; (2) between 40 and 65 years old; (3) being in menopause (31); (4) not involved in any regular exercise program for the last 6 months; (5) completed cancer-related therapies including surgery, chemotherapy and/or radiotherapy at least 6 months prior to enrolling; (6) currently undergoing hormone therapy (tamoxifen or aromatase inhibitor); (7) received medical clearance for exercise training. Patients were excluded from the study if they had musculoskeletal limitations that could compromise exercise performance and/or any uncontrolled chronic disease that could represent a risk to their health.

The study was approved by Research Ethics Committee of the Federal University of Goias (CAAE: 50717115.4.0000.5083), and by the Research Ethics Committee of the Clinical Hospital of the Federal University of Goias (CAAE: 50717115.4.3001.5078). All participants provided written consent.

### Procedures

**Figure 1:**
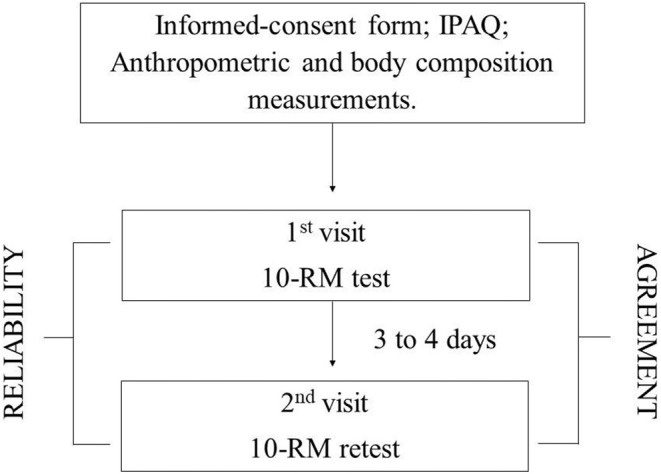
Experimental design. IPAQ, International Physical Activity Questionnaire; 10-RM, 10-repetition maximum.

After a measure of body composition, the participants answered medical history and sociodemographic questionnaire and the International Physical Activity Questionnaire (IPAQ—short version) (32). They then performed the 10-RM test at 2 different days within 2– 4 days in between. At day 1, the participants were familiarized with Leg press 45° and Bench press exercises and then performed the 10-RM test (Figure 1).

#### Anthropometry and Body Composition Assessments

Body mass index (BMI) was calculated based on body mass and height [BMI = weight (kg)/height squared (m^2^)]. Fat and lean mass were assessed using dual energy X-ray absorptiometry (DXA) (General Electric Healthcare® model, Madison, WI, USA). Data were analyzed using GE Medical Systems Lunar™ software. A professional technician performed the assessments of DXADuring the DXA, participants remained in a supine position with their lower limbs relaxed, and the upper limbs were positioned along the body with forearms pronated. DXA's were calibrated and tested as recommended by the manufacturer. After analysis of the entire body area, the total body mass, lean body mass and fat mass were registered.

#### Ten Repetition Maximum Test

The 10-RM test and retests were performed by the leg press 45° (Rocha, Leg Press 45°, Goias, Brazil) and bench press exercises with free-weight, plate-loaded (Supplementary Material). Both exercises techniques followed the recommendation from the National Strength and Conditioning Association (NSCA) (33). During the 10-RM test and retest, the participants were informed and supervised by two experienced exercise science professionals. The same exercise science professionals supervised the measurements. The participants had three to five 10-RM attempts for each exercise.

The warm-up consisted of one set of 10 repetitions with 50% of the estimated 10-RM load, by rating of perceived exertion 5–6 (0–10) in the first day. For leg press, the warm-up represented ~30–40% of their body mass. For bench press, we chose to use only the weight of the barbell (the barbell weighted 6 kg) to perfume the warm-up on the first day. The load used to perform the warm-up during the 10-RM retest was based on the maximum load achieved on the first day (10-RM test).

The 10-RM load was determined if they were able to complete the 10th repetition but not be able to perform the 11th repetition. If the volunteer were able to performed more than 10 repetitions, the load was increased by 5–10%. The resting interval between each attempt was 3 min, and the resting interval between exercises was 5 min. The cadence was not controlled, but participants were oriented to perform the concentric phase as fast as possible but control the eccentric phase. Leg press 45° was performed first, followed by the bench press. All participants performed the bench press until touching the barbell on the sternum/breast. The 10-RM retest was performed 3–4 days later, using the maximum load achieved on the 10-RM test as reference to perform the first attempt (34).

### Statistical Analyses

Descriptive statistics were presented as mean and standard deviation (SD). The intraclass coefficient correlation (ICC) and coefficient of variation (CV = SD divided by mean of test and retest × 100) was used for evaluation of reliability (35). The ICC form used was a two-way mixed effect, mean of k measurements and consistency agreement (36). The ICC and CV are present as mean and 95% of confidential interval (CI). The analyses of measurement error, absolute and relative, of the 10-RM test and retest was also investigated using the standard error of measurement [(SEM); SEM absolute = SD of the mean test-retest score divided by the square root of 1—ICC; SEM relative = SEM absolute score divided by mean test-retest scores and multiplying by 100] and minimally detectable change [(MDC); MDC absolute = 1.96 × the square root of 2 × SEM; MDC relative = MDC absolute score divided by mean of test-retest scores and multiplying by 100] (37). In addition, the limits of agreement were calculated using a Bland–Altman plot with 95% CIs (38). The Munro's classification of reliability was used to interpret the ICC coefficients: 0.50–0.69 reflects moderate correlation; 0.70–0.89 reflects high correlation; and 0.90–1.00 indicates very high correlation. Statistical analyses were performed using MedCalc Software (version 18.11.6) and Statistical Package for the Social Sciences Software (version 22).

## Results

### Participants

The sociodemographic, cancer treatment status, and anthropometric characteristics of the participants are presented in Table 1.

**Table 1:** Characteristics.

**Characteristics**	***N* = 31**
Age (year)—mean (SD)	54.87 (5.7)
**Education—no. (%)**	
<8 years of the study	15 (48.4)
>8 years of the study	16 (51.6)
**Self-reported race—no. (%)**	
Caucasian	20 (64.5)
Non Caucasian	11 (35.5)
**Occupation—no. (%)**	
Homemaker or cleaner	6 (19.4)
Housewife	16 (51.6)
Nurse	1 (3.2)
Retired	5 (16.1)
Saleswoman	2 (6.5)
Teacher	1 (3.2)
**Marital status—no. (%)**	
Single	7 (22.6)
Married	16 (51.6)
Divorced	4 (12.9)
Widow	4 (12.9)
Arterial hypertension—no. (%)	9 (29)
Diabetes—no. (%)	3 (9.7)
Months since cancer diagnosis—mean (SD)	40.68 (14.8)
**Cancer stage—no. (%)**	
I	10 (32.3)
II	17 (54.8)
III	4 (12.9)
**Breast surgery—no. (%)**	
Lumpectomy	1 (3.2)
Lymphadenectomy	1 (3.2)
Mastectomy and breast reconstruction	1 (3.2)
Mastectomy	13 (41.9)
Mastectomy and quadrantectomy	1 (3.2)
Quadrantectomy	13 (41.9)
Quadrantectomy and breast reconstruction	1 (3.2)
Months since breast surgery—mean (SD)	29.03 (15.4)
Axillary lymph nodes removed—mean (SD)	4.86 (4.3)
Chemotherapy—no. (%)	26 (83.9)
Adjuvant	14 (45.2)
Neoadjuvant	12 (38.7)
Missing data	5 (16.1)
Radiotherapy—no. (%)	28 (90.3)
**Hormone therapy—no. (%)**	
Tamoxifen	27(87.1)
Aromatase inhibitors	4 (12.0)
Self-reported lymphedema—no. (%)	13 (41.9)
**Anthropometry and body composition**	
Weight (kg)—mean (SD)	68.67(11.4)
Height (cm)—mean (SD)	157.08 (6.2)
BMI—mean (SD)	27.85(4.5)
Body fat (%)—mean (SD)	46.36(5.90)
Body fat mass (kg)—mean (SD)	31.18(8.33)
Body lean mass (kg)—mean (SD)	35.26(4.64)
Level physical activity (MET-h/wk)—mean (SD)	23.38 (26.40)

*SD, standard deviation; BMI, body mass index; MET, metabolic equivalent of task*.

### Reliability and Agreement Between Test and Retest of 10-RM

The comparison between 10-RM test and retest showed high to very high reliability for the leg press 45° and bench press. For the leg press 45° and bench press exercises the ICC were 0.98 and 0.94, respectively. CV was below 10% for both exercises. The results of reliability are presented in Table 2.

**Table 2:** Analysis of reliability and agreement between 10-RM test and retest.

**Exercises**	**10-RM test (mean ± SD)**	**10-RM retest (mean ± SD)**	**CV (95% CI)**	**ICC (95% CI)**	**SEM (SEM%)**	**MDC (MDC%)**
Leg press (kg)	74.84 (28.50)	80.97 (29.70)	5.87 (3.19–8.55)	0.98 (0.96–0.99)	4.11 (5.27)	5.62 (7.21)
Bench press (kg)	15.03 (3.79)	16.55 (3.70)	7.27 (4.10–10.45)	0.94 (0.87–0.97)	0.96 (6.08)	2.72 (17.20)

*10-RM, 10-repetition maximum; SD, standard deviation; kg, kilogram; CV, coefficient of variation; ICC, intraclass coefficient correlation; CI, confidential interval; SEM, standard error of measurement; MDC, minimally detectable change; LOALB, limits of agreement lower boundary; LOAUB, limits of agreement upper boundary*.

**Figure 2:**
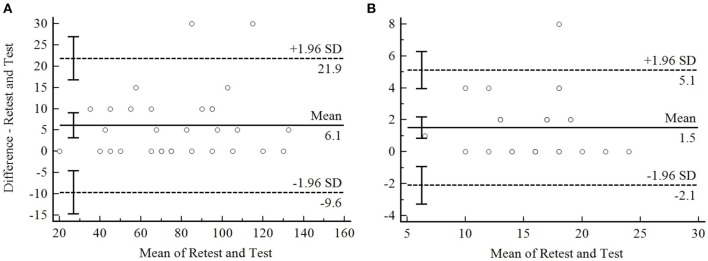
Bland-Altman plot of 10-RM for the leg press 45° **(A)** and the bench press **(B)**. The dotted line represent the limits of agreement upper and lower boundary. The continue line on the center of plot represent the systematic bias. The continue line on the Y axis represent the mean difference between 10-RM retest and test, and on the X axis represent the mean of 10-RM retest and test.

The agreement between the 10-RM test and retest demonstrated that the results from the retest showed higher load than the test situation performed at day 1 (systematic bias values in Figures 2A,B are positives because the analysis were performed with 10-RM retest as first method and 10-RM test as second method for to build the Bland-Altman plots). The Bland-Altman plots (Figure 2) showed the mean difference with 95% IC limits of agreement.

The relative difference between the test and the retest was predicted in 8.3% (limits of agreement for upper and lower boundary 28 and −11%) and 10.3% (limits of agreement for upper and lower boundary 34 and −13%) for the leg press 45° and the bench press, respectively.

The relative and absolute SEM and MDC are presented in Table 2.

## Discussion

This study aimed to evaluate the reliability and agreement between the 10-RM test and retest for the leg press 45° and bench press exercises in BCS. We found a high to very high rate of reliability and agreement with lower and acceptable CV (CV < 10%), SEM (absolute and relative) and MDC (absolute and relative) between the 10-RM test and retest for both the leg press 45°. and bench press However, a higher value was found in the 10-RM retest situation, for both exercises. To our knowledge, this study is the first to evaluate the 10-RM test reliability in BCS, and the results suggest that 10-RM test could be used to measure muscular strength.

In general, a few studies have previously reported the reliability of test and retest 10-RM. In older people, Farinatti et al. (27) described high reliability of the 10-RM test for the dumbbell bench press (ICC 0.90; typical error 1.61 kg) and knee extension (ICC 0.96; typical error 2.01 kg) in elderly healthy women (68 ± 4 years old). Farinatti et al. (39) reported a high ICC for the barbell bench press in young (22 ± 2 years old) and elderly women (69 ± 7 years old) (0.91 and 0.90, respectively). For the leg press 45°, a high ICC (0.99) was reported in young healthy people (24 ± 3 years old) (40). Monteiro et al. (41) also reported a high ICC for the leg press 45° (0.92) and the bench press (0.90) in adult women (37.6 ± 1.7 years old). Our study found a similar reliability to those studies. Therefore, it seems that the 10-RM test reliability for BCS is similar to that of healthy individuals of different ages.

The CV of 10-RM test showed be <10% for lower and upper limbs. That was similar compare to 1-RM in BCS (18). Winter-Stone et al. (18) reported CV of the 6.6 and 7.5% for the leg press and chest press, respectively. We found a similar CV for the leg press 45° and bench press for 10-RM, 5.87 and 7.27%, respectively. Moreover, our results suggest that lower limbs have a better reliability than upper limb exercise, as we hypothesized. It could be explain by lower capacity of lifting for upper limbs compare to lower limbs, this may be result of sides effects of breast cancer treatments.

The 10-RM retest achieved higher load than 10-RM test situation, which may suggest some training effects either in technique or muscle strength of the first exercise test. A repeated strength measurement could provide a process of the learning of task, improving the ability/skill to perform the movement. Bernardi et al. (42) showed that skill acquisition to perform maximal voluntary contraction allows better control of neuromuscular system which could provide higher force generation through the trials. Grosicki et al. (43) also found higher value of 1-RM in the second trial than the first trial of assessment in young adults and older people, women and men, for leg press, leg extension and biceps curl. The same behavior was observed by Amarante do Nascimento et al. (44). They found that the second day of testing was higher than first day, but similar with the third day in 1-RM load for bench press and leg extension in elderly women (65 ± 4 years old) (44). Thus, the muscle strength values could be reached in the second or third trial of measurement.

The 10-RM test could be useful in the real word for prescribing or monitoring the load of the training. The use of percentage of 1-RM test may present a large variability in the number of repetition performance. Grosicki et al. (43) showed that using 60% and 80% of 1-RM test the participants were able to perform 28.8 (±9.2)/23.3 (± 16.3) and 17 (±6.5)/12.8 (±7.8) repetitions in younger and older women, respectively. Hence, the session of training would be high or low effort, if use the percentage of 1-RM test. Therefore, it seems that using the load reached from 10-RM test could be more precisely to prescribe and monitor the number of repetitions during the training session, and that may be one advantages of 10-RM test compared to 1-RM test. Another advantage of 10RM test could be a better perception of safety and acceptance in BCS, since there have been reported knesiohpobia, fear of movement (11, 45). In addition, repetition to failure as 10-RM might be used to predict 1-RM loads for the bench press/chest press (46–48) and leg press 45°/horizontal (47–49), with a low error of measurement. However, we did not investigate the accuracy of the 10-RM load to predict a 1-RM load in BCS. Future studies could investigate the accuracy of the 10-RM test to predict a 1-RM load in BC patients and BCS.

## Strengths and Limitations

The study has important strengths. The tests were supervised by two experienced exercise physiologists/professionals that provided better control of the 10-RM test and guaranteed the safety and confidence for the participants to perform higher load, and the homogeneity of the tests. One limitation of the present study included the lack of assessment of shoulder range of motion during the bench press test. However, we think this limitation was eliminated by the experienced physiologists.

## Conclusion

In conclusion, muscular strength measurement using 10-RM test has a good to excellent rate of reliability and agreement, with acceptable error of measurement. Due to lack of information about the reliability of 1-RM test in BCS, 10-RM test could be an interesting alternative for diagnosis and prescription in this population. Therefore, the 10-RM test may be used to evaluate the muscular strength in BCS. The new studies with BC patients and BCS could report the reliability of the maximum force production on isoinertial exercises.

## Data Availability Statement

The datasets generated for this study are available on request to the corresponding author.

## Ethics Statement

The studies involving human participants were reviewed and approved by Research Ethics Committee of the Federal University of Goias (CAAE: 50717115.4.0000.5083), and by the Research Ethics Committee of the Clinical Hospital of the Federal University of Goias (CAAE: 50717115.4.3001.5078). The patients/participants provided their written informed consent to participate in this study.

## Author Contributions

WS and CV performed the study concept and design. WS and GS supervised the muscle assessments. WS, RS, and WM conducted the analyses. WS wrote the original draft of the manuscript. AV, WM, PG, and CV wrote, reviewed, and edited the manuscript.

### Conflict of Interest

The authors declare that the research was conducted in the absence of any commercial or financial relationships that could be construed as a potential conflict of interest.
