# Supplementary figures and images for: Reliability and Agreement of the 10-Repetition Maximum Test in Breast Cancer Survivors
Source: Front Oncol. 2019 Sep 26;9:918. doi: 10.3389/fonc.2019.00918 (PMC6775190; doi:10.3389/fonc.2019.00918)

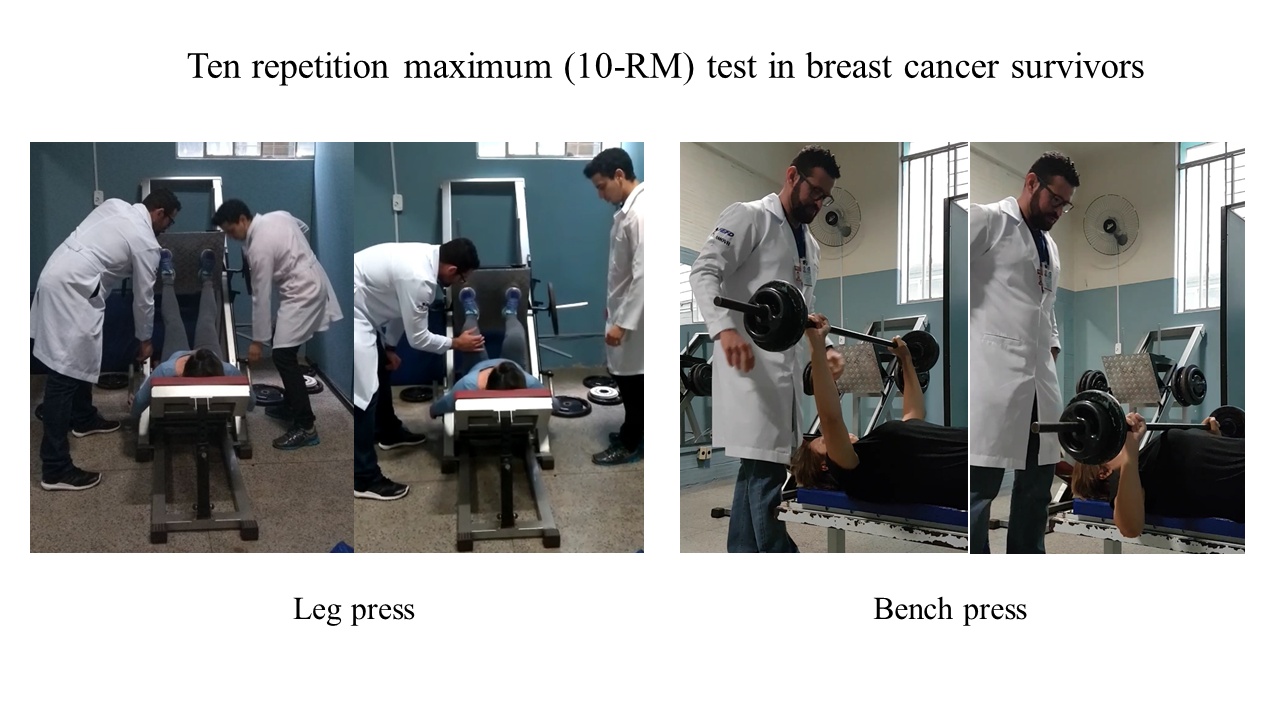

Supplement: Supplementary file 1 [file Image_1.TIF]
